# Brain age modifies the association between blood biomarkers of Alzheimer's disease and cognitive function

**DOI:** 10.1002/alz70856_106538

**Published:** 2026-01-07

**Authors:** Kelsey R. Sewell, Patricio Solis‐Urra, Haiqing Huang, George Grove, Arthur F. Kramer, Edward McAuley, Jeffrey M. Burns, Charles Hillman, Eric D Vidoni, Anna Marsland, Chaeryon Kang, Lu Wan, Lauren Oberlin, Kirk I. Erickson

**Affiliations:** ^1^ Advent Health, Orlando, FL, USA; ^2^ AdventHealth Research Institute, Orlando, FL, USA; ^3^ Faculty of Sport Sciences, Sport and Health University Research Institute (iMUDS), University of Granada., Granada, Granada, Spain; ^4^ AdventHealth Research Institute, Neuroscience, Orlando, FL, USA; ^5^ University of Pittsburgh, Pittsburgh, PA, USA; ^6^ Center for Cognitive & Brain Health, Northeastern University, Boston, MA, USA; ^7^ University of Illinois, Urbana, IL, USA; ^8^ Department of Neurology, University of Kansas School of Medicine, Kansas City, KS, USA; ^9^ Northeastern University, Boston, MA, USA; ^10^ University of Kansas Medical Center, Alzheimer's Disease Research Center, Fairway, KS, USA; ^11^ AdventHealth Neuroscience Institute, Orlando, FL, USA

## Abstract

**Background:**

The development of Alzheimer's disease (AD) involves accumulation of brain pathology; however, some individuals appear to maintain cognitive and day‐to‐day function in the presence of neuropathology better than others. One likely contributor to this resilience is the maintenance of structural brain integrity to a greater extent than expected based on age‐related norms, i.e., a younger ‘brain age’. In this study we examined whether brain age moderated the association between AD‐related blood biomarkers and cognitive function.

**Method:**

We utilized baseline data from the Investigating Gains in Neurocognition in an Intervention Trial of Exercise (IGNITE) study. Cognitively unimpaired older adults (*n* = 648, aged 69.9±3.8, 71% female) completed a comprehensive cognitive assessment and an MRI scan where T1‐weighted images were used to calculate brain age using brainageR. The difference between chronological age and brain age was used to calculate the brain‐predicted age difference (*brain‐PAD*). Assays were completed for plasma‐based biomarkers phosphorylated tau (*p*‐tau) 217 and neurofilament light (NfL) measured on the SIMOA platform.

**Result:**

After covarying for age, sex, site, BMI, image quality and education, *brain‐PAD* moderated the association of *p*‐tau217 with episodic memory (β=‐0.09, *SE* = 0.04, *p* = .017), processing speed (β=‐0.07, *SE* = 0.04, *p* = .046), working memory (β=‐0.10, *SE* = 0.04, *p* = .005), and executive function/attentional control (β=‐0.08, *SE* = 0.04, *p* = .035), but not visuospatial processing. The direction of these interactions was such that the association between higher *p*‐tau217 and poorer cognitive performance was strongest in those with higher *brain‐PAD* (i.e., accelerated brain aging). We do not find evidence of a significant moderation effect of *brain‐PAD* on the association between NfL and cognitive performance in any domain (all *p* >.05).

**Conclusion:**

Our results suggest that maintenance of structural brain integrity (slower brain aging) may help protect against cognitive deficits in the face of AD pathology (i.e., *p*‐tau217) in cognitively unimpaired individuals. These results provide support for the concept of brain maintenance, however, should be further tested in samples across the AD trajectory (i.e., mild cognitive impairment and AD). Understanding these mechanisms could inform strategies to promote resilience and delay AD‐related cognitive decline in aging populations

